# The Insurance Act and Poor-Law Infirmaries

**Published:** 1913-03-08

**Authors:** C. Thackray Parsons

**Affiliations:** Medical Superintendent, Fulham Infirmary.


### THE INSURANCE ACT AND POOR-LAW INFIRMARIES.

By C. THACKRAY PARSONS, M.D.Lond., Medical Superintendent, Fulham Infirmary.

Whilst the National Insurance Act was still under discussion it was generally asserted in the House of Commons and in the public press that one of its results when it came into force would be a great reduction in the number of the sick compelled to obtain medical relief from Poor-Law authorities. Similar views found expression at meetings of many of the Boards of Guardians. On the other hand, most of the Poor-Law officials directly concerned in the administration of relief held that there would be an increase in the number of cases sent into the Poor-Law infirmaries for treatment. Actual experience in the working of the Act since it came into force has amply confirmed this latter view. Many Boards of Guardians are complaining of the number of insured persons sent to them for institutional treatment, and are striving to find means to prevent their admission. There are said to be well over 2,000 insured persons in Poor-Law institutions in London. From inquiries made at some of the infirmaries it would appear that most of the cases sent by the doctors on the panel are cases requiring operation, acute cases needing skilled nursing, such as pneumonia, rheumatic fever, and fractures, or such chronic cases as advanced phthisis, rheumatoid arthritis, ulcers of the leg, and advanced cardiac cases. There can be no doubt that the majority of such cases stand a much better chance of recovery, and that the time during which they are disabled is likely to be considerably reduced by the close medical supervision and skilled nursing they will obtain in the infirmaries. In surgical cases requiring operation the iniquitous provisions of the Act under which the panel doctor has to provide an anæsthetist at his own cost will obviously lead to all operative cases needing anæsthesia being sent to a voluntary hospital or a Poor-Law infirmary, even in the case of the minor operations which have hitherto been undertaken by general practitioners.

Section 12 (2) (iii.) of the Act distinctly states that no persons entitled to maternity, medical, sickness, or disablement benefit shall be refused admission to any Poor-Law infirmary on the ground only of the right to such benefit. The only step Boards of Guardians can take to safeguard themselves against improper use of their institutions is to insist that all cases recommended for admission to their infirmaries by doctors on the panel should be referred to their district medical officers for their opinion as to whether the case could be properly treated at home or not. This procedure has been adopted in most instances. But this places the district medical officer in a difficult position. If he decides the case is not a suitable one for the infirmary he cannot undertake the treatment of the patient himself, but must refer it again to the panel doctor, with the result that a good deal of friction is likely to arise. If, however, the district medical officer decides that it is desirable that the patient should be sent to the infirmary, the Guardians have a distinct grievance in that no part of the sickness, disablement, or maternity benefits can be paid by an approved society to them in respect of maintenance in a Poor-Law institution even if the insured person has no dependants. The Act does, however, provide that any part of the benefit may, if the society or Insurance Committee think fit, be applied in the provision of any surgical appliances required for the insured person or otherwise for his benefit. Under this clause it would seem possible for Guardians to recover the cost of sending an insured person who had been treated in their infirmary to a convalescent home.

It seemed at first as if difficulties would arise in the provision of medical attendance upon the insured resident officers of infirmaries and workhouses, as the Surrey Insurance Committee refused to allow the Medical Superintendent of the Croydon Poor-Law Infirmary to go upon the panel for the purpose of attending only the insured resident officers of that institution. The Croydon Guardians, however, appealed to the Insurance Commissioners, who decided that the resident doctors might go on the panel for this limited service if the other doctors on the panel agreed to divide amongst themselves all the insured persons who made no choice of doctor or were rejected. In London the Insurance Committee have from the first allowed the medical superintendents of Poor-Law infirmaries to join the local panels for the purpose of attending the resident insured officers only, without any proviso at all.

